# Postmortem muscle protein degradation in humans as a tool for PMI delimitation

**DOI:** 10.1007/s00414-016-1349-9

**Published:** 2016-03-07

**Authors:** Stefan Pittner, Bianca Ehrenfellner, Fabio C. Monticelli, Angela Zissler, Alexandra M. Sänger, Walter Stoiber, Peter Steinbacher

**Affiliations:** 1 Department of Cell Biology, University of Salzburg, Hellbrunnerstr. 34, 5020, Salzburg, Austria; 2 Department of Forensic Medicine and Forensic Neuropsychiatry, University of Salzburg, Ignaz-Harrer-Straße 79, 5020, Salzburg, Austria

**Keywords:** Postmortem interval (PMI), Human, Skeletal muscle, Protein, Degradation

## Abstract

Forensic estimation of time since death relies on diverse approaches, including measurement and comparison of environmental and body core temperature and analysis of insect colonization on a dead body. However, most of the applied methods have practical limitations or provide insufficient results under certain circumstances. Thus, new methods that can easily be implemented into forensic routine work are required to deliver more and discrete information about the postmortem interval (PMI). Following a previous work on skeletal muscle degradation in the porcine model, we analyzed human postmortem skeletal muscle samples of 40 forensic cases by Western blotting and casein zymography. Our results demonstrate predictable protein degradation processes in human muscle that are distinctly associated with temperature and the PMI. We provide information on promising degradation markers for certain periods of time postmortem, which can be useful tools for time since death delimitation. In addition, we discuss external influencing factors such as age, body mass index, sex, and cause of death that need to be considered in future routine application of the method in humans.

## Introduction

One of the most important questions at a crime scene and in forensic laboratories is “When did the person die?” A most precise estimate of the time since death is one of the central aspects of forensic science. This is necessary to gain crucial information about the circumstances of death and is, in criminal cases, essential for the confirmation or invalidation of alibis and ultimately for the solution of a crime. Since decades, forensic scientists have investigated changes in postmortem body composition to characterize different phases of decomposition within the postmortem interval (PMI) [1].

The earliest alterations after death (i.e., within the first 24 h postmortem, hpm) include the development and regression of in *rigor mortis* (stiffening and relaxation of skeletal muscles), the progression of *livor mortis* (the settling of blood products in lower parts of the body), and *algor mortis* (the adjustment of the body to environmental temperature) [2]. Further approaches to characterize changes within the early postmortem phase include pharmacological excitability of the eye and electrical stimulation of certain muscles [3–5]. Although these methods are applied to delimitate the PMI in everyday forensic work, there are still great inaccuracies and limitations in many cases.

After several days or even weeks postmortem, it is especially forensic entomology (the analyses of life stages of cadaver-feeding insects) that has shown to be able deliver valuable information about the time since death [6]. However, this method is highly dependent upon the local fauna, the (seasonal) weather conditions, and exposure of the body. Other, more elaborate methods for investigation of the later postmortem phases are colorimetric measurement of tooth pulp [7] and analysis of abdominal and superficial microbial mats [8, 9].

A most prominent lack of reliable methods exists especially for PMI determination in the intermediate phase (i.e., between 24 h and approximately 7 days postmortem). Various studies using animal models have aimed to characterize certain changes in this phase. Especially, degeneration processes in soft tissue (such as brain, heart, lung, liver, kidney, and muscle) have been found to occur within this period of time [10]. However, despite that a large number of studies were able to identify such changes, only very few appear to be practicable or accurate enough to become established for everyday forensic work [1].

Degradation processes of soft tissues have already been described on behalf of other aspects, such as the development of tenderness in stored meat [11–13]. Especially, the analysis of the disintegration of structural muscle proteins using biochemical techniques such as Western blotting or zymography revealed distinct changes in protein band appearance within a certain period of time postmortem [14]. Interestingly, such alterations were reported among proteins in different species of domestic animals in similar fashion (all above), allowing to hypothesize that these patterns also occur in human muscle tissue.

There are several reasons why skeletal muscle is a promising candidate tissue for use in PMI delimitation. Muscle is the most abundant tissue of the human body and is easily accessible while being relatively well protected by the skin. A large number of its proteins are very well characterized, and numerous antibodies for the identification of such proteins are commercially available. Compared to internal organs and nervous tissue, skeletal muscle has a greater delay in postmortem changes but still decomposes faster than cartilage and bone [10]. Together, this makes muscle tissue well suited for routine analysis in the forensic lab and a promising candidate for the analysis of changes postmortem.

In a previous study, we have described degradation processes of several skeletal muscle proteins and enzymes in the pig model [14]. Some of these proteins degraded in a regular and predictably time-dependent fashion, thus making them promising candidates for PMI delimitation also in humans. This was tested in the present work, using samples of human skeletal muscle from 40 forensic cases in Salzburg/Austria. Western blot analysis was employed to examine the degradation behavior of cardiac troponin T, desmin, and tropomyosin. Casein zymography served to determine the inactive-active transitions of calpain 1 and calpain 2. To make additional allowance for temperature, the most important physical factor influencing degradation processes, the data are expressed in accumulated degree-days (ADDs; mean ambient temperature × PMI). As there is still no research available on the relationship between ADD and muscle protein degradation, this study aims to investigate specifically this aspect, together with other possible influencing factors, such as age, sex, body weight, and cause of death, to provide a basis for future routine application of the method in humans.

## Materials and methods

### Subjects

A total of 40 forensic cases were analyzed in this work (23 female and 17 male). To attain minimal inaccuracy, the work was confined to cases with well-known times since death and comprehensible ambient temperature profiles. The subjects were of ages between 2 and 90 years (mean 69.1 ± 19.5 years), with a body mass index between 10.7 and 50.4 (mean 26.2 ± 6.1). Causes of death were diverse and included 27 cases of internal malfunction and organ failure, 10 cases of external trauma, 1 case of intoxication, and 2 unknown causes of death. From a legal point of view, 26 subjects died naturally, 8 in accidents, 2 were suicides, 2 homicides, and 2 were unclear cases. PMI, the time between death and sampling, ranged between 4.0 and 92.8 h postmortem (mean 37.7 ± 27.8 hpm). Twenty eight of the cases were cooled to 4 °C according to Central European standards, with a mean cooling time of 39.1 ± 26.3 h for these cases (Table 1).

**Table 1:** Summary of data collected and calculated for all 40 forensic cases (23 female, 17 male) in the present study

	Mean/SD		Minimum/maximum	
Age	69.1	19.5	2.0	90.0
BMI	26.2	6.1	10.7	50.4
Ambient temperature *T*_*a*_ (°C)	19.8	4.6	−7.0	21.0
*T*_*a*_ below 0 °C, *n* = 3 (hpm)	0.8	0.5	0.5	1.5
4 °C cooling, *n* = 28 (hpm)	39.1	26.3	0.5	88.0
PMI (hpm)	37.7	27.8	4.0	92.8
PMI range (hpm)	0.4	0.9	0.0	3.5
ADD (°d)	10.4	7.7	2.6	36.0

Age data are taken from police or medical records. Body height and weight were measured at the autopsy room and used to calculate the body mass index (BMI = weight [kg]/height^2^ [m]). Values of ambient temperature (*T*_*a*_) were derived from police records or from the nearest meteorological monitoring station. Cooling times were documented at the Forensic Medicine Department of the University of Salzburg. Information on postmortem intervals (PMIs) and ranges of variation were taken from police or medical records. PMI range is used as a measure of PMI value accurateness. ADD values were calculated as follows: PMI × *T*_*a*_

### Accumulated degree-days

ADDs are defined as the product of time and ambient temperature. ADDs have already been successfully employed in animal models and humans to predict postmortem change in corpse morphology [15], DNA degradation [16], and insect colonization [17]. Analysis of postmortem tissue degradation in forensic cases frequently suffers from the limitation that there is no control of environmental conditions. Given this precondition, the use of ADD offers a valuable approach to account for both of the most important influencing factors of postmortem degradation processes, time and temperature, in a standardized manner. For this purpose, the PMI of each case, as measured in days, was multiplied with the respective environmental temperature (in °C). Known phases of different temperature within the PMI (e.g., times of cooling to 4 °C) were separately calculated and the results added to obtain the final ADD value. For dead bodies discovered outside, information on environmental temperature was taken from police records if available or was estimated using the data of the nearest meteorological station. An ambient temperature of 0 °C was considered the lower threshold to prevent negative values. ADD calculation for all 40 cases resulted in a mean of 10.4 ± 7.7 with a minimum of 2.6 °d and a maximum of 36.0 °d.

### Muscle sampling and processing

In all cases, a piece of muscle tissue with a size of approximately 2 × 2 × 2 cm was removed from the lateral thigh muscle (*Musculus vastus lateralis*) in a depth of 2 cm. Excised muscle samples were sectioned to smaller pieces of approximately 100 mg, snap frozen, and stored in liquid nitrogen. Frozen tissue was homogenized by cryogenic grinding and sonication. For casein zymography, an extraction buffer containing 50 mM Tris, 5 mM EDTA, and 10 mM 3-mercaptopropane-1,2-diol was used. For SDS-PAGE and Western blotting, RIPA buffer with a protease inhibitor cocktail was used as extraction buffer. The homogenate was centrifuged at 1000×*g* for 6 min, and the supernatant was removed and stored until further use. Protein concentration was measured using Pierce BCA Assay Kit.

### SDS-PAGE and Western blotting

SDS-PAGE was performed according to Laemmli [18]. Ten-percent polyacrylamide resolving gels (acrylamide:N,N′-bis-methylene acrylamide = 37.5:1, 0.1 % SDS, 0.05 % TEMED, 0.05 % APS, and 375 mM Tris-HCl, pH 8.8) were used for the detection of tropomyosin, desmin, and cTnT. Five-percent polyacrylamide gels (acrylamide:N,N′-bis-methylene acrylamide = 37.5:1, 0.1 % SDS, 0.125 % TEMED, 0.075 % APS, and 125 mM Tris-HCl, pH 6.8) were used as stacking gels. Thirty microgram of total protein was diluted in 15 μl *Aqua bidest* and 5 μl sample buffer (40 % glycerine, 10 % mercaptoethanol, 0.04 % bromphenol blue, and 250 mM Tris-HCl, pH 6.75). Samples were then denatured at 90 °C for 5 min and inserted into the gel wells. Electrophoresis was run at a constant voltage of 150 V until the dye front reached the bottom of the gel (approximately 1.5 h). The running buffer contained 25 mM Tris, 195 mM glycine, 100 mM EDTA, and 0.1 % SDS. Proteins were transferred onto polyvinylidene fluoride (PVDF) membranes in transfer buffer (192 mM glycine, 20 % methanol, and 25 mM Tris) at a constant current of 250 mA for 75 min. All blots were blocked for 1 h in TTBS (150 mM NaCl, 0.05 % Tween, and 25 mM Tris, pH 7.5) including 1 % dried milk as a blocking agent. The following primary antisera were used: mouse monoclonal anti-tropomyosin, mouse monoclonal anti-desmin, and mouse monoclonal anti-cardiac troponin T. HRP-conjugated polyclonal goat anti-mouse was applied as secondary antibody. All antibodies were diluted in a 1 % dried milk solution in TTBS and applied for 1 h. After each antibody application, the membranes were extensively washed and rinsed in TTBS. Antibody binding was visualized by application of chemiluminescence substrate and photographed using a digital gel documentation system.

### Casein zymography

Casein zymography was performed in accordance to the method of Raser [19] with slight modifications. Of polyacrylamide gels (acrylamide:N,N′-bis-methylene acrylamide = 37.5:1, 0.1 % TEMED, 0.05 % APS, and 375 mM Tris-HCl, pH 8.8), 12.5 % were copolymerized with 0.1 % casein and used as resolving gels. Five-percent polyacrylamide gels (acrylamide:N,N′-bis-methylene acrylamide = 37.5:1, 0.125 % TEMED, 0.075 % APS, and 330 mM Tris-HCl, pH 6.8) were used as stacking gels. Two-part sample supernatants were mixed with one-part sample buffer (25 % glycerol, 0.1 % bromphenol blue, and 62.5 mM Tris-HCl, pH 6.8) and one-part *A. bidest*. The running buffer contained 25 mM Tris, 192 mM glycine, and 1 mM EDTA. After a 15-min prerun at 75 V and 4 °C, the samples were inserted into the gel wells and electrophoresis was run for approximately 7 h at 150 V and 4 °C.

The gels were briefly rinsed with *A. bidest*, transferred to incubation buffer (0.1 % 3-mercapto-1,2-propanediol and 50 mM Tris-HCl, pH 7.5) with 4 mM CaCl_2_, and incubated overnight (12–18 h at room temperature). The gels were stained in Coomassie dye (0.1 % Coomassie Brilliant Blue R250, 50 % methanol, and 10 % acidic acid) for 1 h, de-stained in the dye solvent, and photographed using a digital gel documentation system.

### Data interpretation and statistical analyses

Protein band intensities were measured using the gel analysis tools of ImageJ software (v.1.48 NIH, National Institutes of Health, USA); histograms of the tonal distribution of the images were plotted and areas underneath the graphs were measured according to the program’s standard protocol. All signals <1 % of the respective dominant band were considered background (i.e., not regarded a protein band). This provided the basis for obtaining binary information on the presence (1) or absence (0) of proteins, the target variable of the study. All data pairs consisting of information on the presence of an individual protein (1/0) and the respective ADD value were then analyzed for bivariate correlations. Spearman’s *ρ* and *p* values were determined to test whether protein presence is random within the ADD range investigated (Table 2). Significant correlations were further described using logistic regressions. This allows relating the presence probability of a protein band to ADD, thus enabling to determine the ADD at which a specific degradation product can be expected to be present in a significant amount of cases (e.g., 95 %). All statistical analyses were performed using SPSS 22 (IBM, USA).

**Table 2:** The results of protein degradation analyses within the total group of cases, as well as—when meaningful—age- and BMI-restricted groups

	Group	Number			Correlation with ADD		Presence probability (°d)	
		Total	Absent	Present	Spearman’s *ρ*	*p* Value	*P* = 50 %	*P* = 95 %
cTnT dp1	Total	40	3	37	Few cases		n.d.	
cTnT dp2	Total	40	18	22	0.552	0.000	9.4	28.2
	Age <80 and >18	24	10	14	0.851	0.000	7.3	12.6
	BMI <30 and >19	24	11	13	0.637	0.001	7.1	12.4
desmin dp1	Total	40	16	24	0.500	0.002	6.7	28.1
	Age <80 and >18	24	10	14	0.715	0.000	7.7	15.4
	BMI <30 and >19	24	12	12	No significant correlation		n.d.	
desmin dp2	Total	40	19	21	No significant correlation		n.d.	
	Age <80 and >18	24	11	13	0.567	0.007	9.0	23.3
	BMI <30 and >19	24	14	10	No significant correlation		n.d.	
desmin dp3	Total	40	32	8	Few cases		n.d.	
calpain 1 dp1	Total	40	26	14	0.491	0.002	15.0	39.6
	Age <80 and >18	24	14	10	0.668	0.001	11.0	21.2
	BMI <30 and >19	24	16	8	0.520	0.010	12.8	27.5

The first column depicts the number of cases with a certain degradation product (dp). Given that two groups (absent vs present dp) provide large enough sample size, the possible correlation with the ADD was determined using Spearman’s *ρ* (second column H1, there is a correlation between ADD value and the presence (1) or absence (0) of degradation products, and H0, presence of degradation products is random within ADD values). Logistic regression curves were determined for all degradation products with significant correlation to the ADD (*p* ≤ 0.01). The third column represents the predicted presence probabilities (*P*) of 50 and 95 % of the logistic regression

## Results

### Protein analysis

**Fig. 1:**
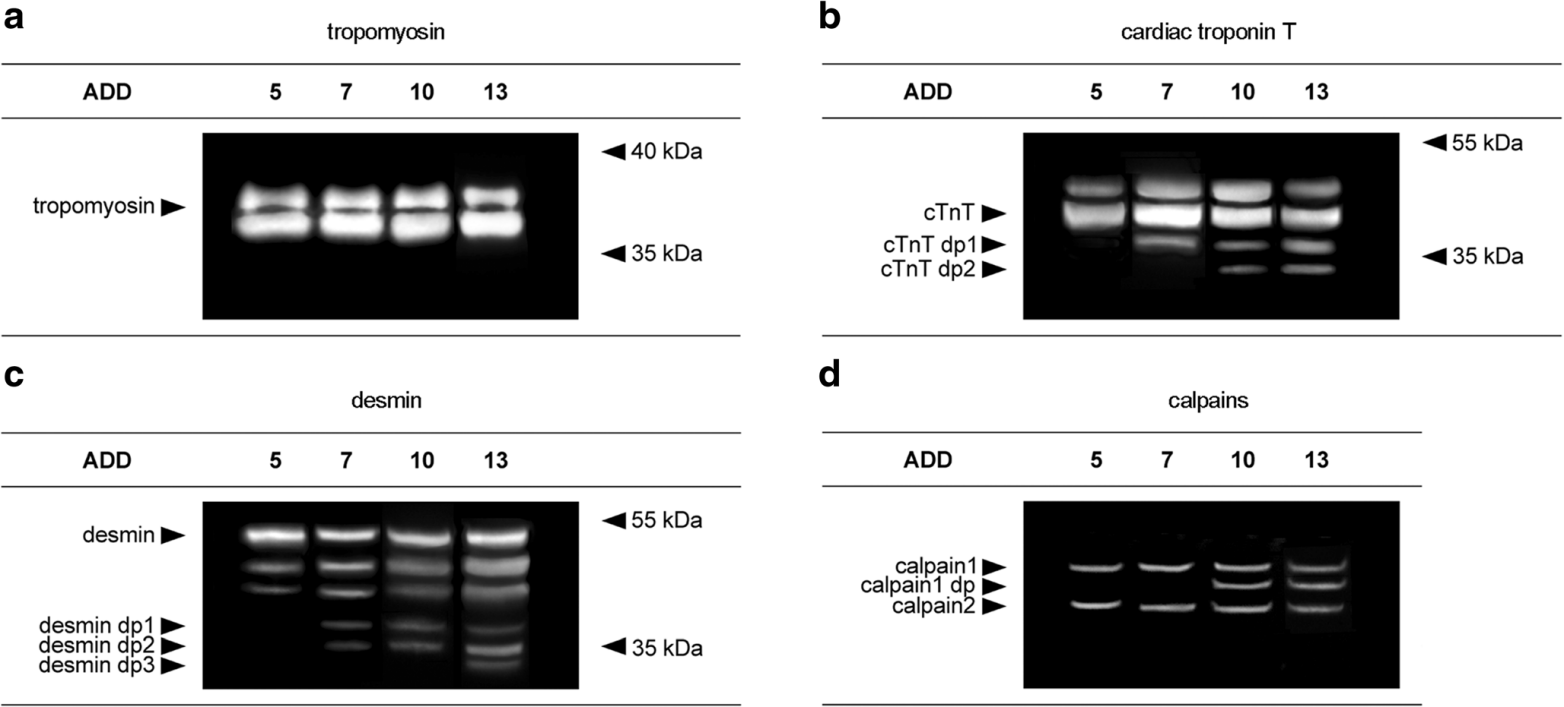
Degradation behavior of tropomyosin, cTnT, desmin, and calpain of four individual cases with varying ADD. Western blot (**a–c**) and zymography (**d**) analyses of muscle protein degradation. Tropomyosin bands (**a**) remain stable independent of ADD and no degradation products are found. By contrast, degradation products (dp) of cTnT (**b**), desmin (**c**), and calpain 1 (**d**) appear in samples that exceed a certain ADD. The protein profiles shown originate from four individual muscle samples but are exemplary for all cases investigated

All samples analyzed exhibited a characteristic tropomyosin double band at approximately 36 to 38 kDa, depicting two isoforms of tropomyosin (Fig. 1). There was no appearance of tropomyosin degradation products or lack of a native band detected in any of the samples.

**Fig. 2:**
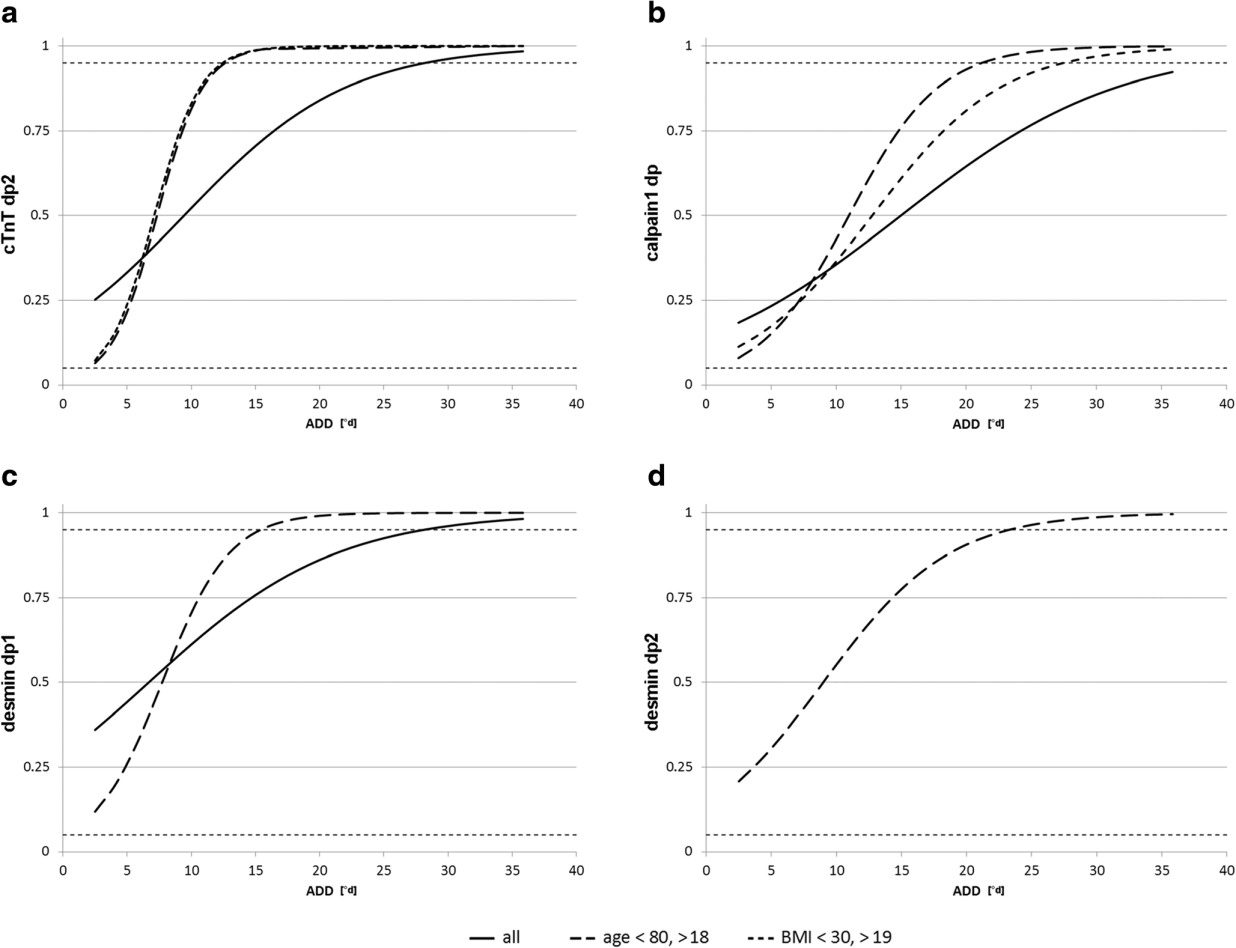
Logistic regression curves of significantly ADD-correlated degradation products cTnT dp2 (**a**), calpain 1 dp (**b**), desmin dp1 (**c**), and desmin dp2 (**d**) represent presence probability development. Regression curves are plotted within the ADD range from 2.6 to 36.0. *Solid lines* stand for the regression of the total group (40 samples), whereas *broken lines* represent age-corrected (*dashes*) and BMI-corrected (*dots*) groups. With increasing ADD, the presence probability of all degradation products rises. Especially, the age-corrected regressions are steep in all cases and exceed the *P* = 0.95 confidence limit (*dotted horizontal line*) at lower ADD compared to the total group

Similarly, analysis of cTnT revealed a band doublet between 40 and 50 kDa in all investigated cases. However, in 37 of the 40 cases, an additional band, most probably representing a cTnT degradation product (cTnT dp1), was detected at approximately 38 kDa. The small number of cases without that particular extra band prevented a statistical correlation with ADDs. In addition, a second degradation product (cTnT dp2) with a molecular weight of about 33 kDa was found below the cTnT dp1 band in 22 cases. The correlation between the presence of the cTnT dp2 band and ADD is highly significant (Spearman’s *ρ* = 0.552, *p* ≤ 0.001). Logistic regression analysis reveals that cTnT dp2 is more likely to be present than absent from 9.4 °d onward (inflection point of curve) and significantly present from 28.2 °d onward (>95 % likelihood of band presence; Fig. 2).

Desmin Western blots showed a band triplet between 45 and 55 kDa in all but one cases. The only exception was the sample of a 77-year-old male with a PMI of 3.7 °d which rendered only a single desmin band. Many of the samples showed additional bands representing desmin degradation products, desmin dp1 at about 38 kDa in 24 cases, desmin dp2 at 35 kDa in 21 cases, and desmin dp3 at approximately 32 kDa in 8 cases (Fig. 1). Although these degradation products seemed to appear consecutively (in no sample, dp2 was found in the absence of dp1 or dp3 in the absence dp1 and dp2), there was no statistical correlation found between the presence of desmin dp2 and dp3 and the ADD. By contrast, desmin dp1 was found to be significantly correlated with ADD (*ρ* = 0.500, *p* ≤ 0.01). This degradation product was significantly present from 28.1 °d onward (in 50 % of the cases at 6.7 °d).

A native PAGE on casein-copolymerized gels with subsequent zymography was performed to obtain insight into the postmortem activity of the calpain system. Once activated in incubation buffer, two bands were detected in all samples. These bands are known to represent calpain 1 and calpain 2 in a native state [19]. An additional band (here termed calpain dp1) localized between the bands of calpain 1 and 2 could be detected in 14 cases. This band is described to represent an autolyzed and hence activated form of calpain 1 [20]. Its presence could be highly significantly correlated with the time since death (*ρ* = 0.491, *p* ≤ 0.01). Regression analysis reveals that this degradation product is present in half of the cases and in 95 % of the cases at 15.0 and 39.6 °d, respectively. No activated form of calpain 2 was detected in any of the 40 cases.

### Influencing factors

Apart from the effects of temperature, there is little information on the influencing factors of protein degradation processes in human muscle. Aiming to reduce this deficit, we investigated the possible influence of the factors age, BMI, cause of death, and sex on postmortem protein decomposition.

#### Age

For this purpose, we performed, in addition to analysis of the total group of cases, a statistical analysis in an age-corrected group, excluding individuals below 18 and above 80 years. This was done to evaluate whether the potential presence of developmental isoforms of muscle proteins in young persons or changes related to severe sarcopenia in very old persons influences the correlation between protein degradation data and the ADD. Correlation coefficients reveal that the degradation of all investigated proteins correlates stronger with the ADD in the age-corrected group than in the total group (Table 2). This includes that the second degradation product of desmin (desmin dp2), which does not correlate with ADD in the total group, shows a significant level of correlation when tested against the age-corrected group (Spearman’s *ρ* = 0.567, *p* ≤ 0.01). In addition to correlation analysis, logistic regression models were fitted for each degradation product in the age-corrected group. Regression curves showed distinctly steeper slopes in all cases, indicating smaller time windows for the appearance of degradation products (Table 2 and Fig. 2). Thus, cTnT dp2 and desmin dp1 become significantly present (*P* > 95 %) at about half of the ADD value compared to the total group (12.6 vs 28.2 °d for cTnT dp1 and 15.4 vs 28.1 °d for desmin dp1). Desmin dp2 and calpain dp1 exceed 95 % probability of presence at 23.3 and 21.3 °d, respectively.

#### BMI

In a second evaluation, we examined groups that were corrected on the basis of body mass index, with similar results as obtained with age-corrected groups. Exclusion of all cases with a BMI higher than 30 (value widely considered as the limit of obesity) and below 19 (considered as underweight or even cachectic) results in increased correlation coefficients compared to the total group for cTnT dp2 and calpain 1 dp1. This was confirmed by logistic regression, with curves becoming steeper in both cases. The presence probability of cTnT dp2 exceeds 95 % at 12.4 °d, compared to 28.2 °d when using all samples, and calpain dp1 reaches 95 % probability of presence at 21.2 °d compared to 39.6 °d. By contrast, exclusion of overweight and underweight cases did not improve the correlation values of both desmin degradation products.

#### Cause of death

Attempts to identify cause of death as influencing factor turned out to be complex. The 40 subjects examined had over 20 different causes of death diagnosed, thus making it virtually impossible to cluster them into groups, large enough to allow sensible comparison. When causes of death are classified into the four categories, “internal” (27 cases), “trauma” (10), “intoxication” (1), and unknown (2), evidently, only the categories internal and trauma are large enough for attempting further analysis. Correlating these causes of death with age expectedly revealed a highly significant relationship. Internal cases correlate positively with age (Spearman’s *ρ* = 0.502, *p* ≤ 0.001), and trauma cases negatively (*ρ* = −0.494, *p* ≤ 0.001), making it impossible to determine cause of death effects in an uninfluenced manner.

#### Sex

Cases were assigned accordingly to analyze sex as a further possible influencing factor. No major influence of sex on the correlation with ADD was detected for troponin (female Spearman’s *ρ* = 0.566, male *ρ* = 0.561) and calpain 1 degradation products (female Spearman’s *ρ* = 0.513, male *ρ* = 0.561). By contrast, the correlations for the presence of both degradation products of desmin were clearly increased in the female group (desmin dp1 *ρ* = 0.662, desmin dp2 *ρ* = 0.407) but decreased in the male group (desmin dp1 *ρ* = 0.361, desmin dp2 *ρ* = 0.195), both compared to the total group.

## Discussion

The results of this study clearly demonstrate the capability of muscle protein analyses to serve as a novel method for the delimitation of the time since death in humans. Degradation processes of skeletal muscle are identified to exhibit a discrete dependence upon accumulated degree-days, as a measure of the postmortem interval. Specifically, we were able to identify particular proteins (desmin, cTnT, and calpain 1) and their degradation products that can be used as markers for specific time intervals in the postmortem decomposition of a human body.

The desmin degradation products of humans are similar to those found in meat science studies, analyzing molecular composition of muscle proteins in various domestic animals such as cattle [21], pigs [22], lamb [23], and chicken [24]. These split products of desmin appeared regularly from 1 to 2 days postmortem onward, and their development was shown to be promoted by incubation of the muscle tissue with calpain 1 and calpain 2 [25, 26]. Similar desmin degradation products were also detected in our previous study of postmortem muscle alteration in pigs [14]. In this work, we monitored desmin degradation products over a period of 10 days. Comparison to the present human data indicates a later appearance of some of these split products in the porcine muscle. This accounts, for example, for the 38-kDa desmin degradation product (desmin dp1), which is significantly present from 28.1 °d onward in humans but did not arise before 56.5 °d in pigs. Whether this divergence results from interspecific differences in the protein itself, or as a consequence of the different physical “storage” conditions (e.g., regarding humidity), remains to be determined. Interspecific variation in the degradation velocity of the desmin protein may be an explanatory factor. However, information on desmin degradation time is limited and suggests that it occurs rather slowly. In cattle muscle, a complete degradation of the native desmin band has not been found before 112 °d [12]. Because the maximum ADD of the present cases in far below this value, the present work does not yield further improvement in this respect.

A high degree of similarity with our previous study in pigs [14] is also noted for the results on the decomposition of cTnT. This relates specifically to the observations that degradation products in pigs and humans are the same in size and that their presence is clearly associated with the time since death. The finding that the appearance of cTnT dp1 does not correlate with the ADD is most probably due to the small number of cases (3 out of 40), in which this degradation product was not detected (Table 2). However, the observation that two of the three cases without that band had a rather short ADD of 2.6 and 3.1 °d might provide a hint that cTnT dp1 emerges soon after death. The third case, a person with an ADD of 15.5 °d and a disproportionately high BMI of 50.4, interestingly presented a band for cTnT dp2 (indicating advanced cTnT degradation) while lacking a band for cTnT dp1.

In human muscle, particularly, cTnT dp2 correlates significantly with the ADD. Exhibiting the highest correlation coefficient of all protein derivatives analyzed, cTnT dp2 presents itself as a most valuable decomposition marker in PMI analysis. Similar to desmin, the cTnT degradation products appeared earlier in humans than in pigs (e.g., cTnT dp2 at 28.2 and 125.7 °d, respectively). Temperature- and PMI-dependent degradation kinetics of cTnT have recently been found in explanted human heart tissue [27]. Note that cardiac troponin T is not limited to cardiac muscle but also occurs in skeletal muscle tissue [25].

As a further result of the present work, it was demonstrated that some proteins remain unaffected by degradation processes throughout the observed period. This accounts for tropomyosin which was present as a double band in all analyzed cases. This is in agreement with our findings on porcine muscle protein degradation in the first 10 days postmortem [14]. However, as tropomyosin is also known to be a substrate for calpain proteolysis [28], it is a candidate for split product occurrence in more advanced stages of muscle decomposition stages.

A particular focus in the present work was placed on the patterns of calpain activation. Enzymes of the calpain family are known to play a major role in muscle protein degradation [29]. They are responsible for proteolytic degradation of a variety of proteins, including desmin, tropomyosin, and cTnT [30]. The presence of the autolyzed and hence activated form of calpain 1 in the investigated human muscle samples may therefore be a direct cause for the observed degradation patterns of desmin and cTnT in these samples. By contrast, there was no autolyzed form of calpain 2 until 36 °d. This is in agreement with our previous data from porcine muscle, demonstrating that activation of calpain 2 is substantially delayed compared to calpain 1 (100.6 vs 18.9 °d) [14]. It may be hypothesized that this delay is evoked by the different Ca^2+^ sensitivity of the two isoforms. It is much higher for calpain 1 than for calpain 2 [29]. The present data demonstrate that the activation of calpain 1 itself is also dependent upon PMI and temperature and significantly occurs at 39.6 °d. This complies with results from Western blot analyses in bovine, ovine, and porcine muscles, showing that the 80-kDa subunit of calpain 1 almost entirely degraded into an active 76-kDa subunit within the first 7 days postmortem, while the 80-kDa subunit of calpain 2 remained un-degraded in this period [14, 23, 31]. Thus, the activity profiles of these enzymes also characterize specific postmortem phases, making them promising candidates for PMI delimitation.

In addition to identifying indicator proteins for future use in time-precise PMI determination, the findings of this study contribute to extend the present understanding of postmortem muscle decomposition generally and of how they are influenced by environmental and demographic factors. Comprehensive knowledge of these aspects is crucial for the development of a protein degradation-based method for PMI analysis and essential to define excluding factors of the method and to determine the scope of the estimation.

The most important external element influencing body decomposition is temperature. Showing clear correlations between the degradation of skeletal muscle proteins and ADD, the present data confirm the usefulness of ADD as a reference measure in PMI delimitation. In addition, the data provide evidence that age and body mass are also important factors that influence postmortem muscle protein degradation. Exclusion of extreme cases (i.e., very old or very young and obese or underweight/cachectic, respectively) definitely improves the predictive precision of the proposed method (Table 1). This is also clearly evident from the steeper slopes of the curves rendered by the regression models for the age- and BMI-corrected groups. Considerations as to the reasons of the age and BMI effects drive attention to a variety of aspects.

Regarding age, the differences in muscle protein degradation may be attributed to either the presence of developmental isoforms in young or often pronounced muscle wasting (i.e., sarcopenia) in the very old subjects [32]. Interestingly, an analysis of the postmortem rise in vitreous potassium levels also demonstrated that subjects below 18 and over 80 years substantially deviate from the age groups in between [33].

It is generally accepted that body mass influences the progression of *algor mortis* because loss of temperature occurs more slowly in bodies with high masses than in cachectic ones. Thus, the BMI is applied as an important correction factor in this regard [1]. The influence of body mass on protein degradation observed in the present work may, on the one hand, be a direct consequence of the above described differences in body cooling rates. On the other hand, it may also be associated with the finding that subjects with very high and very low BMI have sarcopenic symptoms and abnormal protein metabolism rates [32, 34]. A similar BMI-dependent influence has been demonstrated for RNA integrity in muscle tissue after death, which is significantly lower in samples from persons with a BMI > 25 [35].

In contrast to age and body mass, sex was found to exert no major effects on postmortem protein degradation. The only exception is the ADD correlation of the two degradation products of desmin, which is clearly lower in males than in females. The underlying mechanism of this difference remains unclear, as well as to whether there is any relation to the fact that desmin is one of only a few muscle proteins with higher expression levels in men than in women [36].

In summary, the present study demonstrates that analysis of muscle protein degradation processes has a high potential for being a useful tool in forensic PMI delimitation. We identify candidate proteins with degradation properties to make them most suitable for delimiting certain periods of time postmortem, even under the heterogeneous conditions that are inevitably encountered when examining non-standardized human subjects. This offers a chance to establish a new method for the delimitation of the time since death. The proposed approach provides two important advantages: (i) Muscle tissue is highly abundant in human bodies; it is easily accessible during forensic examination and contains a vast set of proteins, each with the capability to act as a marker molecule for different stages postmortem. (ii) Protein analysis by gel electrophoresis and Western blotting is an easy method, established in almost every biochemical laboratory, and delivers discrete information about degradation processes. Several proteins can be simultaneously tested within 24 h, and a huge variety of antisera for muscle proteins is commercially available. Thus, this method has the potential to support and eventually substitute complicated or less accurate existing approaches. However, additional research is still needed to amplify the group of marker proteins. So far, we present two lower limits for ADD intervals with 95 % probability in an overall approach, as well as four lower 95 % limits considering demographic restrictions for this method. Also, it is still necessary to fine-tune the correction for the bias introduced by factors such as age and body mass. Moreover, specifically, longitudinal studies allowing repeated sampling of individual subjects through an extended period of time postmortem would offer the chance to obtain valuable additional information about the exact point of time of certain degradation phenomena.
